# Periosteal Dry Needling for Carpometacarpal Osteoarthritis: A Prospective Case Series

**DOI:** 10.3390/jcm12175678

**Published:** 2023-08-31

**Authors:** Anna Staehli Wiser, James Dunning, Casey Charlebois, Paul Bliton, Firas Mourad

**Affiliations:** 1 Redington Fairview General Hospital, Skowhegan, ME 04976, USA; 2 American Academy of Manipulative Therapy Fellowship in Orthopaedic Manual Physical Therapy, Montgomery, AL 36104, USA; 3 Montgomery Osteopractic Physical Therapy & Acupuncture, Montgomery, AL 36106, USA; 4 William S. Middleton VA Hospital, Madison, WI 53705, USA; 5 Department of Physiotherapy, LUNEX International University of Health, Exercise and Sports, 4671 Differdange, Luxembourg; 6 Luxembourg Health & Sport Sciences Research Institute A.s.b.l., 50, Avenue du Parc des Sports, 4671 Differdange, Luxembourg

**Keywords:** dry needling, osteoarthritis, carpometacarpal joint, thumb pain, periosteal, CMC OA

## Abstract

Background: Carpometacarpal (CMC) osteoarthritis (OA) of the thumb is a painful condition that affects over 15% of individuals above the age of 30 and up to 30% of post-menopausal women. Dry needling (DN) has been found to reduce pain and disability in a variety of neuromusculoskeletal conditions; however, DN in the management of CMC OA has not been well studied. Methods: Consecutive patients with clinical and radiographic evidence of CMC OA were treated with DN. The primary outcome measure was pain using the Numerical Pain Rating Scale (NPRS) at 12 weeks. Secondary outcome measures were the Upper Extremity Functional Index (UEFI-20) and the Global Rating of Change (GROC) scale. Outcome measures were collected at baseline, 4 weeks, 8 weeks, and 12 weeks. Results: Nine patients were treated for six sessions of periosteal DN over 3 weeks. Compared to baseline, statistically significant and clinically meaningful improvements were observed in thumb pain (NPRS mean difference: 2.6; *p* = 0.029) and function (UEFI-20 mean difference: 21.3; *p* = 0.012) at 12 weeks. Conclusion: Statistically significant and clinically meaningful within-group improvements in thumb pain and function were observed at 12 weeks following six sessions of periosteal DN treatment. DN may be a useful intervention in the management of patients with CMC OA of the thumb.

## 1. Introduction

### Background

Carpometacarpal (CMC) osteoarthritis (OA) of the thumb is a debilitating hand-wrist joint condition that affects 15% of individuals over the age of 30 and up to 30% of post-menopausal women [1,2]. CMC OA thumb pain limits gripping, grasping, and dexterity activities, making several common functional activities difficult to perform, such as holding a cup, combing hair, carrying objects, or gripping a steering wheel.

A primary cause of CMC OA is thought to be associated with radial subluxation from the deterioration of the anterior oblique and dorsoradial ligaments of the thumb, leading to joint incongruence, radial subluxation, erosion of the articular cartilage, inflammation, pain, and joint stiffness [3]. Compensatory hyperextension of the metacarpophalangeal (MCP) joint is often seen as the condition progresses [4].

The diagnosis of CMC OA is based on radiographic findings and a clinical examination [1,2,5]. The clinical signs of CMC OA include a positive “hump” sign (a large lump over the CMC joint), a positive grind test, joint crepitation, swelling, point tenderness, weakness, and the inability to abduct the thumb. Carpal tunnel syndrome, De Quervain’s tenosynovitis, lateral epicondylitis, trigger thumb, and radial nerve entrapment are common co-existing conditions often seen with CMC OA. Current conservative treatments include bracing, activity modification, cortisone injection, and occupational and/or physical therapy [1]. 

Several studies seem to associate the etiology of musculoskeletal pain with poor circulation [6,7]. DN uses the insertion of solid filiform needles into the areas of neuromuscular tissue dysfunction to elicit acute tissue damage and, in turn, create a healing response by increasing blood flow to an area [8]. Notably, DN has been found to be effective in reducing pain, stiffness and/or disability associated with OA in the knee and upper extremity by improving circulation to the joint and surrounding tissues (muscle, nerve, bone, cartilage) [9,10,11,12,13,14,15,16,17,18,19].

Clinically meaningful improvements in pain and disability have been reported in a number of trials where periosteal needling—i.e., moving the needle close to the bone, joint line, or cartilage, or tapping the needle repeatedly onto the bone—was utilized in patients with hip or knee OA [20,21,22,23]. Notably, electroacupuncture appears to stimulate cartilage repair in individuals with knee OA. More specifically, following 20-min sessions over 4 weeks of 7-point, low-frequency electroacupuncture, Zhang et al. [24] reported significantly lower T2 values on magnetic resonance imaging at the anteromedial and anterolateral tibial subregions in 100 knees of 50 patients with knee OA. 

In addition, interleukin-6 mRNA expression in bone marrow has been found to diminish following acupuncture (i.e., needling without injectate), thereby limiting inflammation and inhibiting the myelogenic osteoclast activity driving osteoarthritic degeneration [25]. Moreover, acupuncture may improve joint lubrication through changes in the hyaluronic acid within the synovial fluid [26]. Notably, acupuncture (i.e., periarticular needling without injectate) has also been found to increase microcirculation to the knee joint [10,11]. In addition, electroacupuncture has been found to block the release of local inflammatory cytokines in the synovia of osteoarthritic joints [27] and block the release of systemic inflammatory factors in the periaqueductal gray of the brain stem [28]. 

A 2018 multi-center randomized clinical trial of 242 patients with knee osteoarthritis found the inclusion of periosteal electrical dry needling to be more effective for their pain and disability reduction than manual therapy and exercise alone [29]. However, to date, there is conflicting and limited evidence for the use of DN in individuals with thumb pain associated with OA of the CMC joint. A recent randomized controlled trial of patients with basal thumb pain found no between-group difference in pain following six visits of verum acupuncture compared with sham acupuncture [30]; however, a recent case study found significant reductions in thumb pain following DN [31]. Notably, current systematic reviews do not include acupuncture or dry needling as a conservative treatment option for thumb pain [1,32,33]. The purpose of this case series was to explore the use of DN as an invasive but conservative treatment option in patients with CMC OA joint pain. This case report follows the CARE checklist [34]. 

## 2. Case Description

### 2.1. Patients

Consecutive patients who presented to the outpatient physical therapy clinic at Redington-Fairview General Hospital, Skowhegan, Maine, between August and December 2022 with a doctor’s prescription to treat thumb pain were screened for inclusion. Their ages ranged between 49 to 73 years, with a mean (SD) of 60.9 (7.7) years. The duration of symptoms ranged from 12 months to 60 months, with a mean (SD) of 29.3 (20.9) months. Baseline characteristics of the nine patients in this case series can be found in Table 1. 

The inclusion criteria were as follows: (1) primary complaint of thumb pain lasting longer than 3 months, (2) pain with movement of the thumb, (3) limited hand function due to thumb pain, and (4) clinical evidence of CMC osteoarthritis (i.e., a positive grind test and point tenderness at the joint line). A positive grind test has been found as a valid and reliable test for the clinical diagnosis of symptomatic CMC OA [35], with a sensitivity of 0.42, specificity of 0.96, and an interrater reliability of 0.48 [36,37] 

The exclusion criteria were as follows: (1) had received a steroid injection to the thumb within the past 3 months, (2) had prior surgery to the thumb, (3) had received physical or occupational therapy treatment for thumb pain within the previous 3 months, (4) had evidence of cervical radiculopathy or referred pain from the cervical spine, (5) had one or more contraindications to dry needling, including red flags (i.e., tumor, fracture, metabolic diseases), (6) a history of mastectomy or lymph node removal involving the ipsilateral side, (7) were currently pregnant, (8) had surgery involving the ipsilateral upper extremity within the past year, and (9) had pending legal action or a worker’s compensation claim regarding their thumb pain. All patients who met the criteria and agreed to participate underwent a formal informed-consent process by signing a consent form approved by the Institutional Review Board at Redington-Fairview General Hospital, Skowhegan, Maine, prior to the physical therapy evaluation. 

### 2.2. Treating Clinician

The physical therapist who examined and treated all patients had 20 years of clinical experience and was a fellow in training within the American Academy of Manipulative Therapy Fellowship in Orthopaedic Manual Physical Therapy program. The clinician had 4 years of experience in dry needling, including a 30 h APTA-accredited course in dry needling and a 54 h dry needling certification training course that included content specific to the thumb. 

### 2.3. Evaluation Procedure

For each patient, a thorough physical examination was conducted to verify the clinical signs of CMC osteoarthritis. This examination included a visual inspection for nodules, joint enlargement and swelling, palpation for tenderness at the joint and surrounding soft tissues, and compression/distraction special tests for joint pain and crepitus. Screening measures were performed, as indicated, to identify any co-existing injuries of the upper extremity or cervical spine. Measures of strength and the active range of motion were also recorded.

### 2.4. Outcome Measures

Patients completed the Numeric Pain Rating Scale (NPRS) and the Upper Extremity Functional Index-20 (UEFI-20) at the initial evaluation and 4, 8, and 12-week intervals following the baseline examination. The primary outcome measure was the NPRS at 12 weeks. The NPRS was used to measure the thumb pain intensity. Patients reported the average intensity of thumb pain over the past week using an 11-point scale ranging from 0 (no pain) to 10 (worst pain imaginable) at baseline, 4 weeks, 8 weeks, and 12 weeks following the initial treatment session [38]. The NPRS has been found to be a reliable and valid instrument for the assessment of pain intensity [39,40,41]. The MCID for the NPRS has been reported to be 1.74 in patients with chronic musculoskeletal pain conditions [41]; however, the MCID for thumb-related pain has not yet been established. Nevertheless, a change of 2 points or a 30% decrease in pain from baseline has previously been considered the MCID in patients with chronic musculoskeletal pain conditions [41,42].

The secondary outcome measures included the Upper Extremity Functional Index (UEFI-20) and Global Rating of Change scale (GROC). The UEFI-20 was recorded at baseline, 4 weeks, 8 weeks, and 12 weeks. The UEFI-20 has been found to possess strong construct validity and high inter-examiner reliability [43,44]. Scores range from 0 to 80, with higher scores indicating higher function. Patients rated their self-perceived changes in function using a 15-point GROC questionnaire based on a scale described by Jaeschke et al. [45]. The MCID for the GROC has not been established; however, scores of +4 and +5 are considered indicative of moderate changes in patient status [45].

### 2.5. Intervention 

All participants received DN 2 x per week for 3 weeks for a total of 6 treatments.

Previous needling studies have used one needle inserted into the dorsal mid thenar eminence into the belly of the adductor pollicis brevis, several acupoints around the CMC joint line, and into the extensor carpi radialis longus and along the radial nerve pathway but the needles were not placed into the key muscles in the thenar eminence (opponens pollicis, abductor pollicis brevis, flexor pollicis brevis).

**Figure 1:**
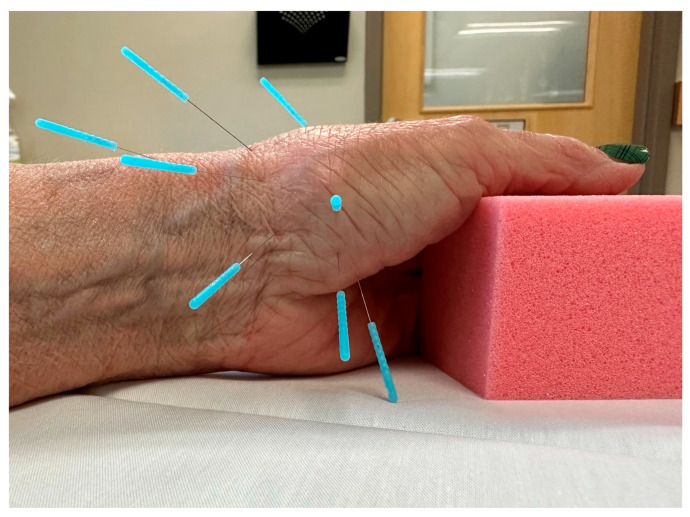
Standardized dry needling protocol (8 needles) for CMC OA.

For this study, a standardized dry needling protocol was used to ensure consistency (Figure 1, Table 2). 

The standardized dry needling protocol included eight points that targeted muscle, periarticular, and perineural tissue of the affected thumb. Needles were inserted to approximate the anatomical target, then rotated to achieve myofascial tenting and elicit a *deqi* response (i.e., a dull ache, heaviness, spreading, distention, or warmth) [46]. Unidirectional needle rotation is a recommended technique for eliciting myofascial release and a chemical healing cascade [47]. The needles were re-manipulated if necessary every 5 min and were left in situ for a total of 20 min. Two sizes of Seirin J type neeldes were utlizied (0.18 × 15 mm and 0.25 × 30 mm). 

### 2.6. Treatment Side Effects

The patients were asked to report any adverse events they experienced during the study. An adverse event was defined as sequelae of a 1-week duration, perceived as distressing and unacceptable to the patient that required further attention [47]. Particular attention was given to ecchymosis and post-needling soreness. Aside from minor and temporary bruising and soreness, no adverse events were reported.

### 2.7. Statistical Analysis

Data analysis was performed using SPSS 28.0 (Chicago, IL, USA). Descriptive statistics, including frequency counts for the categorical variables and measures of central tendency and dispersion for continuous variables, were calculated to summarize the findings. A normal distribution for the NPRS and UEFI-20 was assessed using the Kolmogorov–Smirnov test; both outcomes were normally distributed (*p* > 0.05). A one-way analysis of variance (ANOVA) for repeated measures, with a Greenhouse–Geisser epsilon correction, was used to compare the within-group scores over time for each continuous variable (1 for the NPRS data and 1 for the UEFI-20 data). Post hoc pairwise comparisons were performed to examine the difference between the baseline and each of the follow-up periods using the Bonferroni correction at an α level of 0.05. The statistical analysis was conducted at a 95% confidence level. All nine participants completed the outcomes through the 12-week follow-up. To quantify the magnitude of the treatment effect, the within-group effect sizes were calculated using Cohen’s d coefficient. An effect size of greater than 0.8 was considered large, an approximating 0.5 was considered moderate, and less than 0.2 was considered small.

## 3. Results

A single physical therapist screened 11 consecutive patients with thumb pain for eligibility. Of the 11 patients screened, 1 was excluded due to symptoms consistent with carpal tunnel syndrome and possible cervical radiculopathy, and 1 was excluded because their thumb pain was deemed to primarily be associated with dysfunction at the MCP joint. Therefore, nine patients met the inclusion criteria and consented to participate in the study. Nine patients (mean age, 60.9 years; range, 49–73) were treated for six visits over a 3-week period (2 times per week). All patients completed all six treatment sessions. 

### 3.1. Thumb Pain (NPRS)

**Figure 2:**
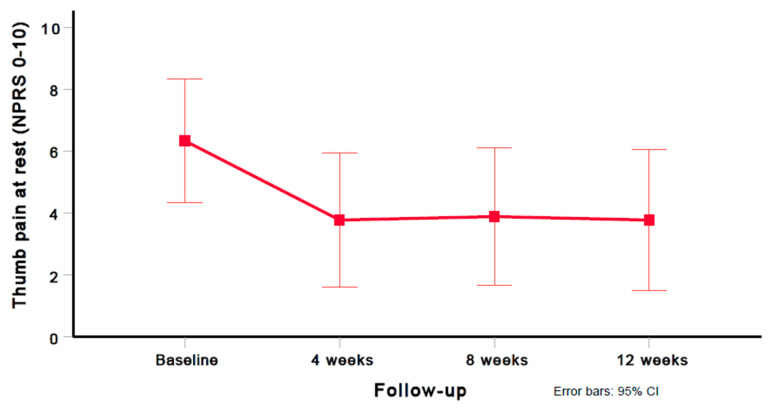
Evolution of thumb pain intensity (NPRS 0–10). Values are mean and 95% confidence interval.

Using the Greenhouse–Geisser epsilon correction, a one-way repeated-measures ANOVA demonstrated a significant (F = 6.779; *p* = 0.024) decrease in thumb pain (NPRS) after six dry needling treatment sessions (Figure 2). 

A significant improvement in thumb pain intensity (NPRS mean difference, 2.6) was observed between the baseline and 12 weeks (*p* = 0.029); however, no significant differences in thumb pain intensity (NPRS) were found between 4 and 8 weeks (*p* = 0.729), and 8 and 12 weeks (*p* = 0.594). Compared with the baseline, large (i.e., Cohen’s *d* ≥ 0.8) within-group effect sizes were observed for thumb pain intensity (NPRS) at 4 weeks (Cohen’s *d* = 0.875), 8 weeks (Cohen’s *d* = 0.939), and 12 weeks (Cohen’s *d* = 0.888). Table 3 provides the mean and standard deviation (SD) for the thumb pain intensity (NPRS) scores at all assessment periods (baseline, 4 weeks, 8 weeks, and 12 weeks). Table 4 provides the preintervention and postintervention scores for thumb pain intensity (NPRS) for each of the subjects at all time points.

### 3.2. Upper Extremity Functional Index (UEFI-20) 

**Figure 3:**
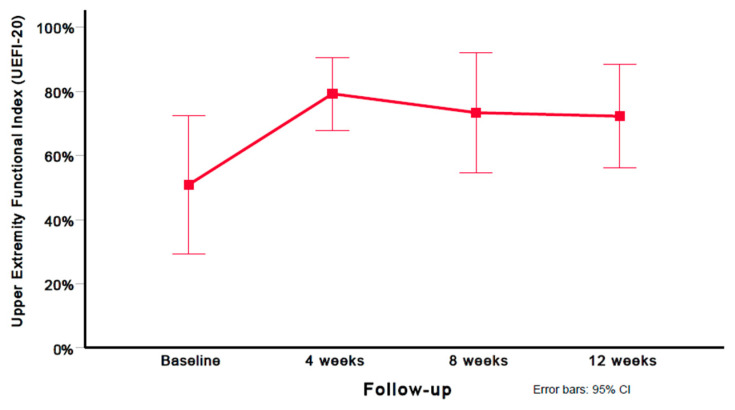
Evolution of disability scores (UEFI-20). Values are mean and 95% confidence interval.

Using the Greenhouse–Geisser epsilon correction, a one-way repeated measures ANOVA found a significant (F = 6.148; *p* = 0.010) decrease in disability (UEFI-20 score) after six sessions of DN (Figure 3). 

A significant improvement in disability (UEFI-20 mean difference: 21.33) was observed between the baseline and 12 weeks (*p* = 0.012); however, no significant differences in disability (UEFI-20) were found between 4 and 8 weeks (*p* = 0.511), and 8 and 12 weeks (*p* = 0.678). Compared with the baseline, large (i.e., Cohen’s *d* ≥ 0.8) within-group effect sizes were observed for disability (UEFI-20) at 4 weeks (Cohen’s *d* = 1.11), 8 weeks (Cohen’s *d* = 1.05), and 12 weeks (Cohen’s *d* = 1.08). Table 3 provides the mean and standard deviation (SD) for the disability (UEFI-20) scores at all assessment periods (baseline, 4 weeks, 8 weeks, and 12 weeks). Table 5 provides the preintervention and postintervention scores for UEFI-20 on each of the subjects at all time points.

### 3.3. Global Rating of Change (GROC)

In nine patients with thumb pain due to CMC OA and after six sessions of DN, the mean (SD) GROC scores were +4.0 (2.1) at 4 weeks, +2.4 (3.0) at 8 weeks, and +2.2 (2.9) at 12 weeks follow-up, indicating “moderately better” and “slightly better” outcomes in the short and medium term, respectively. 

## 4. Discussion

Nine patients with CMC OA were treated for six sessions of periosteal DN over 3 weeks. Statistically significant and clinically meaningful within-group improvements were observed in thumb pain (NPRS mean difference: 2.6; *p* = 0.029) and function (UEFI-20 mean difference: 21.3; *p* = 0.012) at 12 weeks. Notably, most improvement was observed at 4 weeks; however, this improvement appeared to persist following the completion of the treatment regimen at 3 weeks and through the final follow-up at 12 weeks. Four out of the nine subjects exceeded the MCID of a 2 or more points (NPRS 0–10) reduction in pain at 4, 8, and 12 weeks. For the disability, eight out of nine subjects exceeded the MCID of 10% improvement for the UEFI-20 at 4 weeks, and six out of nine subjects met the MCID of 10% improvement at 8 weeks and 12 weeks. In addition, six of the nine patients scored themselves at 4 or better at the 4-week interval, indicating that more than 60% of the patients reported a “moderate” improvement in their condition by the completion of the six sessions of periosteal dry needling to the CMC joint. 

Barnard et al. found no difference between real and sham acupuncture for basal thumb pain [30]; in addition, Dickens and Lewith found no significant improvement in thumb pain following acupuncture in patients with CMC OA [48]. Nevertheless, unlike the current case series, periosteal needling targeting the bone, joint line, and periarticular connective tissue of the CMC joint was not utilized in the two prior studies [30,48]. Notably, a recent multi-center clinical trial of 242 patients with knee osteoarthritis found that the addition of periosteal electrical dry needling was more effective for pain and disability reduction than manual therapy and exercise alone [29]. Likewise, a 2021 case study reported significant reductions in thumb pain following DN [31].

The underlying mechanisms as to why the patients receiving DN around the CMC joint experienced a reduction in pain and an improvement in function remain to be elucidated. However, a number of studies have found periosteal needling—i.e., moving the needle close to the bone, cartilage, or joint line—leads to clinically meaningful improvements in pain and disability in patients with hip and/or knee OA [20,23,49]. More specifically, following 20-min sessions over 4 weeks of low-frequency electroacupuncture, Zhang et al. [24] reported significantly lower T2 values on MRIs at the anteromedial and anterolateral tibial subregions of 100 knees, suggesting electroacupuncture may play a role in the cartilage repair in individuals with knee OA. Notably, acupuncture has been found to diminish interleukin-6 mRNA expression in bone marrow, thereby reducing inflammation and inhibiting myelogenic osteoclast activity driving degeneration [25]. Additionally, increased levels of hyaluronic acid within the synovial fluid appear to be induced by acupuncture, thus enhancing joint lubrication [26]. Interestingly, local electroacupuncture has been found to enhance joint microcirculation at the knee [10,11]; furthermore, electroacupuncture has been found to block the release of local inflammatory cytokines (i.e., interleukin-1 β and tumor necrosis factor-α) in the synovia of osteoarthritic joints [27] and block the release of systemic inflammatory factors in the periaqueductal gray matter of the brain stem [28].

### Limitations

Although a cause-and-effect relationship cannot be inferred from the results of a case series, clinically meaningful within-group changes in pain and function were observed; nevertheless, these changes may be due to natural history, changes in activity levels, the Hawthorn effect [50], and/or the therapeutic alliance [51]. Additionally, all treatment sessions were administered by a single physical therapist who was completing a postgraduate fellowship program in orthopedic manual physical therapy; therefore, the results may not be generalizable to all clinicians [52].

## 5. Conclusions

This case series suggests that periosteal DN may be a useful intervention for pain and disability reduction in patients with basal thumb pain associated with OA of the CMC joint. Randomized clinical trials are needed to determine the effectiveness and between-group effect sizes when compared to an active comparison or control group. 

## Figures and Tables

**Table 1:** Baseline variables: demographics and outcome measures.

Baseline Variable	Patients with CMC OA (*n* = 9)
Age (y)	60.9 (7.7)
Sex: female, *n* (%)	9 (100%)
Duration of symptoms (months)	29.3 (20.9)
BMI (kg/m^2^)	32.8 (10.9)
NPRS (0–10)	6.3 (2.6)
UEFS (0–100%)	50.9 (28.1)

Data are expressed as mean (SD). NPRS, Numerical Pain Rating Scale, 0–10, lower scores indicate less pain; UEFS-20, Upper Extremity Functional Scale −20, 0–100%, higher scores indicate greater function.

**Table 2:** Standardized 8-point dry needling protocol for CMC OA.

Points	Anatomical Target Tissue	Location	Needle Angulation
Point 1	Adductor pollicis	From a dorsal approach, needle through the first web space, staying close to the border of the first metacarpal	P to A slightly ulnar
Point 2	Opponens pollicis; abductor pollicis brevis; recurrent branch of median nerve; base of CMC	Radial side of first metacarpal, within the proximal third of thenar eminence, close to the first metacarpal. Pass through the abductor pollicis brevis muscle and toward the bone into the opponens pollicis, which lies against the first metacarpal	Needle inserted A to P, angle toward base of CMC
Point 3	Abductor pollicis brevis; base of CMC	Within the proximal third of the thenar eminence, one finger width medial to point 2	Needle inserted A to P, angle toward base of CMC
Point 4	Recurrent branch of median nerve; flexor pollicis brevis; base of CMC	Within the proximal third of the thenar eminence, one finger width medial to point 3	Needle inserted A to P, angle toward base of CMC
Point 5	Joint capsule of CMC; Superficial radial nerve	Within the anatomical snuff box between the tendons of EPL and EPB	Angle toward CMC joint
Point 6	Capsule of the CMC joint; Superficial radial nerve	At the wrist crease on the radial side of the radial artery, under the APL tendon	Slide under the APL tendon
Point 7	Superficial radial nerve; within tendon sheath of APL and EPB	Two finger widths proximal to the center of the anatomical snuff box, just proximal to radial aspect of styloid process of radius between and parallel to tendons of APL and EPB muscles	Tangential insertion by gripping and lifting the tissue; squeeze in using free insertion
Point 8	Palmar aspect of first trapezio-metacarpal joint; median nerve	One finger width distal to the flexion wrist crease on the radial and palmar side at the base of the CMC joint	Perpendicular toward CMC joint

**Table 3:** Preintervention and postintervention scores for shoulder pain, disability, and GROC.

Variable	Preintervention	4 Weeks	8 Weeks	12 Weeks
NPRS (0–10)	6.3 (2.6)	3.8 (2.8)	3.9 (2.9)	3.8 (2.9)
UEFS-20 (0–100%)	50.9 (28.0)	79.2 (14.8)	73.3 (24.5)	72.2 (21.1)
GROC (−7 to +7)	NA	4.0 (2.1)	2.4 (3.0)	2.2 (2.9)

Data are expressed as mean (SD). GROC, Global Rating of Change, −7 to +7, higher scores indicate greater overall improvements; NPRS, Numerical Pain Rating Scale, 0–10, lower scores indicate less pain; UEFS-20, Upper Extremity Functional Scale, 0–100%, higher scores indicate greater function.

**Table 4:** Preintervention and postintervention scores for thumb pain.

NPRS	Preintervention	4 Weeks	8 Weeks	12 Weeks
Subject 1	4	4	3	3
Subject 2	10	10	10	10
Subject 3	6	5	6	6
Subject 4	10	2	4	3
Subject 5	7	3	2	3
Subject 6	8	2	2	1
Subject 7	3	0	0	0
Subject 8	4	3	3	3
Subject 9	5	5	5	5

NPRS, Numerical Pain Rating Scale, 0–10, lower scores indicate less pain.

**Table 5:** Preintervention and postintervention scores for disability.

UEFI-20	Preintervention	4 Weeks	8 Weeks	12 Weeks
Subject 1	36	57	84	76
Subject 2	64	64	64	64
Subject 3	18	68	31	31
Subject 4	20	92	36	50
Subject 5	37	89	84	70
Subject 6	31	68	85	88
Subject 7	82	95	100	95
Subject 8	84	94	90	90
Subject 9	86	86	86	86

UEFS-20, Upper Extremity Functional Scale, 0–100%, higher scores indicate greater function.

## Data Availability

Not applicable.
